# A phase I study of combination vaccine treatment of five therapeutic epitope-peptides for metastatic colorectal cancer; safety, immunological response, and clinical outcome

**DOI:** 10.1186/1479-5876-12-63

**Published:** 2014-03-10

**Authors:** Shoichi Hazama, Yusuke Nakamura, Hiroko Takenouchi, Nobuaki Suzuki, Ryouichi Tsunedomi, Yuka Inoue, Yoshihiro Tokuhisa, Norio Iizuka, Shigefumi Yoshino, Kazuyoshi Takeda, Hirokazu Shinozaki, Akira Kamiya, Hiroyuki Furukawa, Masaaki Oka

**Affiliations:** 1 Department of Digestive Surgery and Surgical Oncology, Yamaguchi University Graduate School of Medicine, 1-1-1 Minami-Kogushi, Ube, Yamaguchi, Japan; 2 Department of Medicine and Surgery, The University of Chicago, 900 E. 57th St., Chicago, IL 60637, USA; 3 Department of Immunology, Juntendo University School of Medicine, 2-1-1 Hongo, Bunkyo-Ku, Tokyo, Japan; 4 Department of Pharmacy, Yamaguchi University Hospital, 1-1-1 Minami-Kogushi, Ube, Yamaguchi, Japan

**Keywords:** Peptide vaccine, Peptide cocktail, Colorectal cancer, Phase I study

## Abstract

**Background:**

To evaluate the safety of combination vaccine treatment of multiple peptides, phase I clinical trial was conducted for patients with advanced colorectal cancer using five novel HLA-A*2402-restricted peptides, three peptides derived from oncoantigens, ring finger protein 43 (RNF43), 34 kDa-translocase of the outer mitochondrial membrane (TOMM34), and insulin-like growth factor–II mRNA binding protein 3 (KOC1), and the remaining two from angiogenesis factors, vascular endothelial growth factor receptor 1 (VEGFR1) and VEGFR2.

**Methods:**

Eighteen HLA- A*2402-positive colorectal cancer patients who had failed to standard therapy were enrolled in this study. 0.5 mg, 1.0 mg or 3.0 mg each of the peptides was mixed with incomplete Freund’s adjuvant and then subcutaneously injected at five separated sites once a week. We also examined possible effect of a single site injection of “the cocktail of 5 peptides” on the immunological responses. ELISPOT assay was performed before and after vaccinations in the schedule of every 4 weeks.

**Results:**

The vaccine treatment using multiple peptides was well tolerated without any severe treatment-associated systemic adverse events. Dose-dependent induction of peptide-specific cytotoxic T lymphocytes was observed. The single injection of “peptides cocktail” did not diminish the immunological responses. Regarding the clinical outcome, one patient achieved complete response and 6 patients revealed stable disease for 4 to 7 months. The median overall survival time (MST) was 13.5 months. Patients, in which we detected induction of cytotoxic T lymphocytes specific to 3 or more peptides, revealed significantly better prognosis (MST; 27.8 months) than those with poorer immune responses (MST; 3.7 months) (p = 0.032).

**Conclusion:**

Our cancer vaccine treatment using multiple peptides is a promising approach for advanced colorectal cancer with the minimum risk of systemic adverse reactions.

**Clinical trial registration:**

UMIN-CTR number UMIN000004948.

## Background

Colorectal cancer (CRC) is the third most common cancer and the second leading cause of cancer-related death in industrialized countries [1]. In the last decade, the combined regimens of multiple anticancer drugs have been applied and markedly improved the survival of patients with CRC at stages III and IV [2]. However, many patients often face to progression of the diseases due to chemo-resistance.

Recent development in genome-based technologies has enabled us to obtain comprehensive gene expression profiles of malignant cells compared with normal cells [3]. By applying cDNA microarray technology coupled with laser micro-dissection, we had identified three oncoantigens, ring finger protein 43 (RNF43) [4], 34 kDa-translocase of the outer mitochondrial membrane (TOMM34) [5], and KOC1 (IMP-3; IGF-II mRNA binding protein 3) [6,7], as targets for development of cancer peptide vaccines for CRC. An oncoantigen was defined as a molecule with high immunogenicity in our immune system and with oncogenic function that plays a critical role in the growth of tumor cells. Since the oncoantigen is essential for the cell growth, the probability of immune escape of cancer cells by reducing or lacking these proteins is expected to be low [8,9]. In addition, these three molecules are specifically expressed in cancer cells, the risk of autoimmune reactions by vaccine treatment using the peptides derived from these proteins is expected to be minimum [4-6].

Although immunotherapy using tumor infiltrating cells (TIL) or vaccine treatment have been expected as a promising modality to treat cancer, recent reports have indicated several mechanisms in tumor tissues to protect cancer cells from immune attacks [10]. For example, the limitation of antitumor effects of cytotoxic T lymphocytes (CTL) was explained by tumor cell heterogeneity; a subset of tumor cells revealed downregulation or loss of expressions of human leukocyte antigen (HLA) or targeted antigen proteins [11,12]. The growth of solid neoplasms always accompanies with neovascularization [13] which is associated with the expression of vascular endothelial growth factor receptor 1 (VEGFR1) [14] and VEGFR2 [15]. These two molecules were highly expressed in tumor vascular endothelial cells. Hence, our vaccine treatment including the peptides derived from VEGFR1 and VEGFR2 is also able to target neovascular endothelial cells, suppress neovascurization, and then reduce the energy and oxygen supply into the tumor tissues.

In this study, since an HLA-A*2402 allele is the most common HLA-A allele in the Japanese population with the allelic frequency of approximately 60% [16], we selected five HLA-A*2402-restricted peptides derived from RNF43, TOMM34, KOC1, VEGFR1, and VEGFR2 for the clinical trial. The purpose of this study was to evaluate the safety and biological responses of these five peptides. Additionally, we compared the immunological responses of the separate injections of each of five peptides with those of the single injection of a cocktail of five peptides. We here demonstrate the safety of these peptides and a promising result of our cocktail treatment of five peptides for the improvement of prognosis of advanced CRC.

## Patients and methods

### Patients and eligibility criteria

The study protocol was approved by the Institutional Ethics Review Boards of Yamaguchi University (H18-82), was carried out in accordance with the Helsinki declaration on experimentation on human subjects, and was registered in the UMIN Clinical Trials Registry as UMIN000004948. Written informed consent was obtained from the patients at the time of enrollment. Patients were eligible for enrollment (1) when they had histologically confirmed CRC without indication of surgical resection, (2) when they had failed to respond to prior standard chemotherapy or were intolerable to the standard therapy, and (3) when they were HLA-A*2402-positive by DNA typing. We monitored for at least 4 weeks from the termination of the prior treatment to the beginning of the vaccine treatment, in order to wait patients’ full recovery from adverse events with grade 3 or higher according to the Common Terminology Criteria for Adverse Events version3.0 (CTCAE). The patients were required to have an Eastern Cooperative Oncology Group performance status (PS) of 0 to 2, to be older than 20 years of age and to have a life expectancy of at least 3 months. Adequate bone marrow function (white blood cell count ≥2,000/mm^3^ and platelet count ≥75,000/mm^3^), renal function (serum creatinine ≤2.0 mg/dl) and liver function (transaminase within 3.0 times the institution's upper limit of normal) were required. Patients were excluded if they were pregnant, had severe ischemic heart disease, had active infectious disease, had any steroid-dependent autoimmune diseases, or had any prior peptide vaccination therapies.

### Peptides

The RNF43-721 (NSQPVWLCL) [17], TOMM34-299 (KLRQEVKQNL) [5], KOC1(IMP-3)-508 (KTVNELQNL) [18], VEGFR1-1084 (SYGVLLWEI) [19] and VEGFR2-169 (RFVPDGNRI) [20] peptides restricted with HLA-A*2402 were synthesized by American Peptide Company Inc. (Sunnyvale, CA, USA) according to a standard solid-phase synthesis method and purified by reverse-phase high performance liquid chromatography (HPLC). The purity (>95%) and the identity of the peptides were determined by analytical HPLC and mass spectrometry analysis, respectively. Endotoxin levels and the bio-burden of these peptides were tested and determined to be within acceptable levels as Good Manufacturing Practice grade for vaccines. These peptides and the epitope peptide derived from the human immunodeficiency virus-envelope (HIV-Env) protein restricted with HLA-A*2402 (RYLRDQQLL) were used to measure the CTL response.

## Study design and End-points

### Study 1

This study was a phase I clinical trial with dose-escalation of the five peptides. This study was primarily conducted to evaluate the safety and to find the recommended dose (RD) of these peptides, and secondarily to evaluate immunological and antitumor effects. Dose escalation was performed in 3 patients’ cohort with doses of 0.5 mg, 1 mg, and 3 mg for each peptide. Each peptide was mixed with 0.5 ml of incomplete Freund's adjuvant (IFA) (Montanide ISA51; Seppic, Paris, France) administered to patients.

### Study 2

Since the theoretical binding affinities of the 5 epitope peptides to HLA-A*2402 were not so different (Table 1), within one order, a single injection of the cocktail of five peptides could be expected to induce immune responses at the same level as separate injections of each of the five peptides. This study was conducted to evaluate the safety as well as immunological and antitumor effects. The cocktail of 5 peptides at the dose of 3 mg was mixed with 1.5 ml of IFA and administered to 6 patients.

**Table 1:** Binding score of each peptide

**Epitope peptides**	**Sequence**	Binding score* to HLA-A*24:02	**Expression in colorectal cancer (%)**	**Remarks on function and tumor relevance**	**References**
RNF43 - 721	NSQPVWLCL	10	90%	Growth of cancer cells	[4,17]
TOMM34 - 299	KLRQEVKQNL	13.4	80%	Growth of cancer cells	[5]
KOC1 - 508	KTVNELQNL	14.4	77%	Metastasis and invasion	[6,7,18]
VEGFR1 - 1084	SYGVLLWEI	66	100%**	Tumor angiogenesis,growth through autocrine	[14,15,19,20]
VEGFR2 - 169	RFVPDGNRI	22			

*The binding affinities were estimated using the BioInformatics and Molecular Analysis Section websites.

**100% in tumor cells as well as tumor associated neovascularity.

### Study 1 & 2

The peptides were administered subcutaneously into the thigh or axilla regions on days 1, 8, 15, and 22 in a 28-day treatment course. Administration of the peptides was performed repeatedly for at least eight weeks for the evaluation of the safety. Vaccination was continued after 8 weeks or even after the progression of the disease when a patient wished and a primary doctor who provided best supportive care or additional chemotherapies, recommended. From the fourth courses of treatment, the vaccination schedule was changed to be biweekly, and from the seven courses, it was reduced to once a month.

A complete blood count and serum chemistry tests were performed every 2 weeks. Signs of toxicity were assessed according to CTCAE. Dose-limiting toxicity was defined as a hematological toxicity of grade 4 or greater and non-hematological toxicity of grade 3 or greater. Fifty milliliters of blood was drawn before each course, and then peripheral-blood mononuclear cells (PBMCs) and blood plasma were isolated. PBMCs and plasma were preserved in liquid nitrogen tank until examination. The vaccinated patients (n = 18) were assessed for immunological and clinical responses according to the Response Evaluation Criteria in Solid Tumors version1.0 (RECIST) as well as serum Carcinoembryonic antigen (CEA). All known sites of disease were evaluated on a monthly basis by computed tomography (CT) or magnetic resonance imaging (MRI) before vaccination and after each course.

### Estimation of local skin reactions at the vaccinated sites

Local skin reactions at injected site of vaccine were assessed according to CTCAE grading.

### Measurement of the peptide-specific IFN-γ response

Antigen-specific T cell response was estimated by enzyme-linked ImmunoSpot (ELISPOT) assays following in vitro sensitization as described previously [21,22]. The number of peptide-specific spots was calculated by subtracting the spot number in the control well from the spot number of a well with vaccinated peptide-pulsed stimulator cells. Antigen specific T cell response was classified into four grades (−, +, ++, or +++) according to the algorithm flow chart described in our previous report (+++: IFN-γ producing cell is contained more than 0.2%, ++: 0.02 - 0.2%, +: 0.01 - 0.02%, −: less than 0.01% in the sample applied for ELISPOT) (Additional file 1: Figure S1 [21]). Sensitivity of our ELISPOT assay was estimated at approximately average level by the ELISPOT panel of the Cancer Immunotherapy Consortium [23].

### Statistical analysis

Overall survival (OS) rates and progression free survival (PFS) rates were analyzed by the Kaplan-Meier method, and survival was measured in days from the first vaccination to succumbing to the disease. P-values were assessed using a log-rank test. Cox regression model was used for multivariate analysis of biomarkers for overall survival. Student's t-test was used for the analysis of peptide specific immune responses. All statistical analyses were performed with SPSS statistics 17.0 (SPSS, Chicago, IL, USA). P < 0.05 was considered statistically significant.

## Results

From February 2007 to March 2009, nineteen CRC patients were enrolled in this study and received vaccine treatment. One patient (case 7) who refused continuation of the vaccine therapy after a single administration of peptides was excluded from this analysis. Characteristics of eighteen patients are summarized in Table 2. Most of the patients except two cases had received chemotherapy regimens including fluorouracil, irinotecan or oxaliplatin prior to the vaccine treatment; one (case 11) of the two exceptions was due to pelvic abscess after the low anterior resection. The other exception (case 16) was because this patient refused chemotherapy. Another patient (case 18) had refused surgical treatment because she was afraid of the high complication risk of the second operation of the recurrent tumor in the pelvis. We provided vaccination with the written informed consent, but she accepted to receive curative resection after 8 weeks of vaccination. This case was included in the immunological analysis, but excluded from the analysis of survival.

**Table 2:** Patients characteristics and outcomes

**Case**	**Dose of peptide (mg)**	**Age**	**Sex**	**PS**	**Site of desease**	Previous treatment*	**IFN γ response to each peptide**					**Number of vaccination**	**Period of vaccinations (days)**	**Toxicity**		**Response**	**Prognosis (days)**			**Treatments after vaccination**
							**R**	**T**	**K**	**R1**	**R2**			**Injection site reaction**	**General**		**PFS**	**OS**		
							**Pre vaccinations**													
							**Within 3 courses of vaccinations**													
							**Within 6 courses of vaccinations**													
1	0.5	56	M	0	LN	IRI, OX, FU	++	–	–	–	–	85	2150	2	None	CR	2150	2150	A	None
							–	–	+	++	–									
							+	++	+	++	–									
2	0.5	72	F	1	Lung, bone	IRI, OX, FU	–	–	–	–	–	16	120	1	None	PD	60	191	D	None
							+	–	–	–	+									
							+	+	–	–	+									
3	0.5	75	M	1	Liver, lung	IRI, OX, FU	–	–	++	–	–	27	364	1	None	SD	158	406	D	OX, FU, BEV
							++	–	–	–	+									
							++	–	+	–	+									
4	1	59	F	2	Dissemi.	IRI, OX, FU	–	–	–	–	–	8	49	1	None	PD	36	80	D	None
							++	–	–	–	–									
							++	–	+	–	+									
5	1	68	M	0	Lung	IRI, OX, FU	–	–	–	–	–	25	329	2	None	PD	68	1009	D	OX, FU, IRI, BEV, CETU
							++	+	++	+	++									
							++	+	++	+	++									
6	1	69	M	2	Lung, LN	IRI, OX, FU	–	–	+	–	–	13	84	2	None	PD	62	110	D	None
							–	++	–	–	–									
							–	++	–	–	–									
8	3	85	F	1	Dissemi.	FU	–	–	–	–	–	11	70	2	None	PD	103	461	D	IRI, FU
							–	–	–	+	–									
							–	–	–	+	–									
9	3	59	M	0	Lung	IRI, OX, FU	+	–	–	–	–	45	777	2	None	SD	221	885	D	None
							++	++	++	++	–									
							++	++	++	++	–									
10	3	49	F	0	Liver, lung	IRI, FU	++	–	–	–	–	39	777	2	Fever <38.0°C	PD	69	834	D	OX, FU, IRI, BEV
							+++	–	++	++	+									
							+++	–	++	++	+									
11	3	46	F	0	Lung	none	–	–	–	–	–	13	64	2	Erythena (G1)	SD	117	1029	D	OX, FU, IRI, BEV
							–	+	++	+++	++									
							–	+	++	+++	++									
12	3	71	M	0	Local	OX, FU	–	–	–	–	–	63	959	2	None	SD	120	1059	D	IRI, FU, OX, BEV
							+	++	++	–	+									
							+	++	++	+	+									
13	3	71	M	2	Liver, lung, bone	IRI, OX, FU, RAD	–	–	–	–	–	8	49	1	None	PD	57	133	D	None
							–	++	++	–	–									
							–	++	++	–	–									
14	3 (mix)	65	F	2	Dissemi.	IRI, OX, FU	–	–	–	–	–	8	49	1	None	PD	50	342	D	None
							+	+	++	+	++									
							+	+	++	+	++									
15	3 (mix)	71	M	2	Liver, lung	IRI, OX, FU	–	–	–	–	–	8	49	1	None	PD	36	102	D	None
							+	–	–	+++	++									
							+	–	–	+++	++									
16	3 (mix)	57	M	1	Local	none	–	–	++	–	–	38	749	2	None	SD	771	1161	D	None
							+	–	–	+++	+++									
							+	++	–	+++	+++									
17	3 (mix)	65	M	1	Dissemi.	OX, FU	–	+	–	+++	–	16	126	2	None	PD	69	145	D	None
							+	+	+	+++	+									
							+	+	+	+++	+									
18	3 (mix)	55	F	0	Local	IRI, FU	–	+	+	–	–	8	49	2	None	SD	56	56	A	Curative resection
							–	–	+	–	–									
							–	–	+	–	–									
19	3 (mix)	58	F	2	Liver, Dissemi.	OX, FU, BEV	–	++	+	–	+	8	49	1	None	PD	37	52	D	None
							++	+	++	–	+									
							++	+	++	–	+									

PS: Eastern Cooperative Oncology Group performance status, LN: lymph nodes, Dessemi: peritoneal dissemination, PFS: progression free survival, OS: over all survival.

IRI: irinotican, OX: oxaliplatin, FU: fluoropyrimidine, RAD: radiation, BEV: bevacizumab, CETU: cetuximab, R: RNF43, T: TOMM34, K: KOC1, R1: VEGFR1, R2: VEGFR2, D: daed, A: alive.

CR:complete response, PR: partial response, SD: stable desease, PD: progression desease, *: operation was performed for all patients for the resection of primary lesions.

### Study 1: dose escalation study

#### Safety, peptides-specific immune responses and recommended dose

The vaccination was well tolerated without any high-grade systemic adverse reactions in any of the 19 patients at any doses as shown in Table 2. However, all patients revealed injection site reactions with grade 1–2 swelling with/without inflammation except two cases with grade 2 ulceration. In this cohort of 19 patients, no dose-limiting toxicity was detected in any patients.

ELISPOT assay (Additional file 2: Figure S2) was performed using the samples obtained before and every 4 weeks after the beginning of vaccinations to evaluate induction of peptide specific CTL by measuring the IFN-γ secretion as biomarkers. The average numbers of specific CTL induction against each of five peptides per each patient within 12 vaccinations (3 courses) at the doses of 0.5 mg, 1.0 mg, and 3.0 mg were 2.0 (6 peptides/3 patients), 2.3 (7/3), and 3.2 (19/6), respectively (Table 3). The CTL responses within 3 courses (12 weeks) of vaccinations were highest in the group who received the dosage of 3.0 mg. We therefore decided that the RD was 3.0 mg/body.

**Table 3:** Number of patients responded to each peptide

**Dose of peptide (mg)**	**Patients**	**R**	**T**	**K**	**R1**	**R2**	**Total**	Average*
	**n**	**Within 3 courses of vaccinations**						
0.5	3	2	0	1	1	2	6	2.0
1	3	2	2	1	1	1	7	2.3
3	6	3	4	5	4	3	19	3.2**
3 (cocktail)	6	5	3	4	4	5	21	3.5**

R: RNF43, T: TOMM34, K: KOC1, R1: VEGFR1, R2: VEGFR2.

*: The average numbers of specific CTL induction against each of five peptides per each patient.

**: There was no significant difference to induce the immune responses between the multiple injections of “each peptide” and the single injection of “the peptide cocktail”. (p=0.694, t-test).

### Study 2: peptide cocktail study

#### Safety and peptides specific IFN-γ response

Since the weekly injection at five independent loci for each peptide is painful to patients and the binding affinities of these 5 epitope peptides to the HLA molecules were suspected to be not so different (Table 1), we examined whether the mixture of five peptides in one shot (cocktail peptide) can induce peptide-specific CTLs at same or similar levels, compared with the five independent shots of each peptide. The cocktail of 3 mg each of 5 peptides was mixed with IFA and tested in 6 patients.

This vaccination protocol was well tolerated without any treatment-associated adverse events except the grade 1 or 2 injection site reaction. We measured peptide-specific CTL responses in these cocktail-treated patients and compared with those in patients who received injection at five independent loci (Table 3). In six “cocktail”-treated patients, we observed induction of CTLs in 5 patients for RNF43, 3 patients for TOMM34, 4 patients for KOC1, 4 patients for VEGFR1, and 5 patients for VEGFR2 (total of 21 peptide-specific CTL inductions in 6 patients) within 3 courses of the vaccination. Among the patients who were injected 3 mg each of the five peptides independently revealed induction of CTLs for RFN43, TOMM34, KOC1, VEGFR1, and VEGFR2 in 3, 4, 5, 4, and 3 patients, respectively (total of 19 peptide-specific CTL inductions in 6 patients). There was no significant difference to induce the immune responses between the multiple injections of “each peptide” and the single injection of “the peptide cocktail” (p = 0.694, t-test). These results indicated that the single injection of “the peptide cocktail” is likely to induce the similar immune responses to the multiple injections of “each peptide”.

### Clinical evaluation

**Figure 1:**
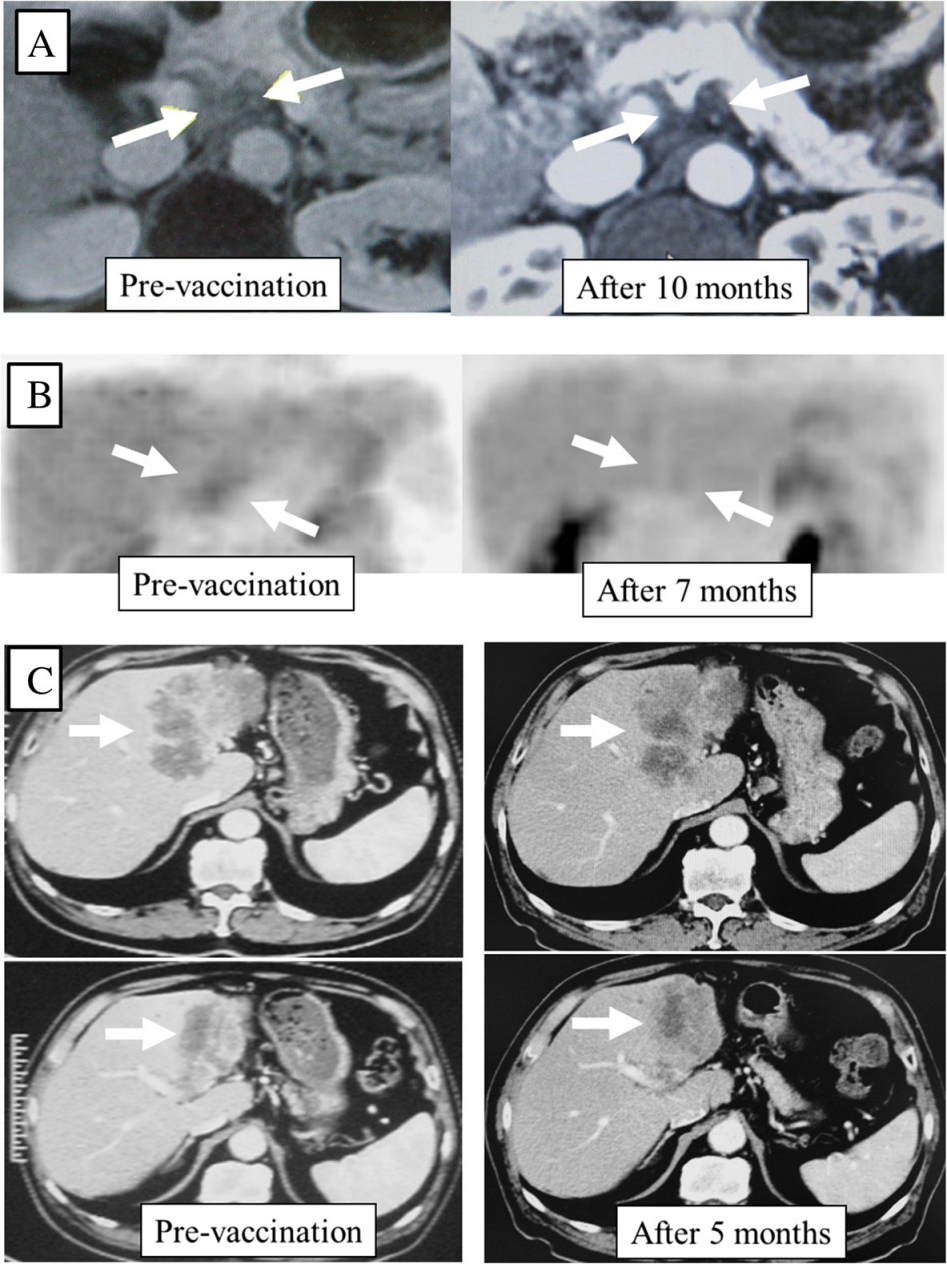
**Diagnostic images of case 1 and case 3. (A)** T1-weighted magnetic resonance imaging (MRI) of representative radiologic response to vaccination in case 1 who achieved complete response. **(B)** Positron Emission Tomography (PET) of case 1. Diffusely infiltrated lymph node metastases around the hepato-duodenal ligament by MRI (**A**, left side) as well as PET (**B**, left side) were confirmed their disappearance by MRI and PET analysis after 10 months of vaccination (**A** &**B**, right side). **(C)** Computed tomography in case 3 who achieved stable disease. The massive liver metastases were kept stably for 5 months.

One patient (case 1) achieved a complete response in lymph node metastasis in the hepato-duodenal ligament and lasted for over 5 years (Figure 1A&B). In addition, 6 patients maintained stable disease condition for 4 to 7 months. In case 3, the massive liver metastases as well as lung metastases (data not shown) were kept stably for 5 months as shown in Figure 1C.

### Survival

**Figure 2:**
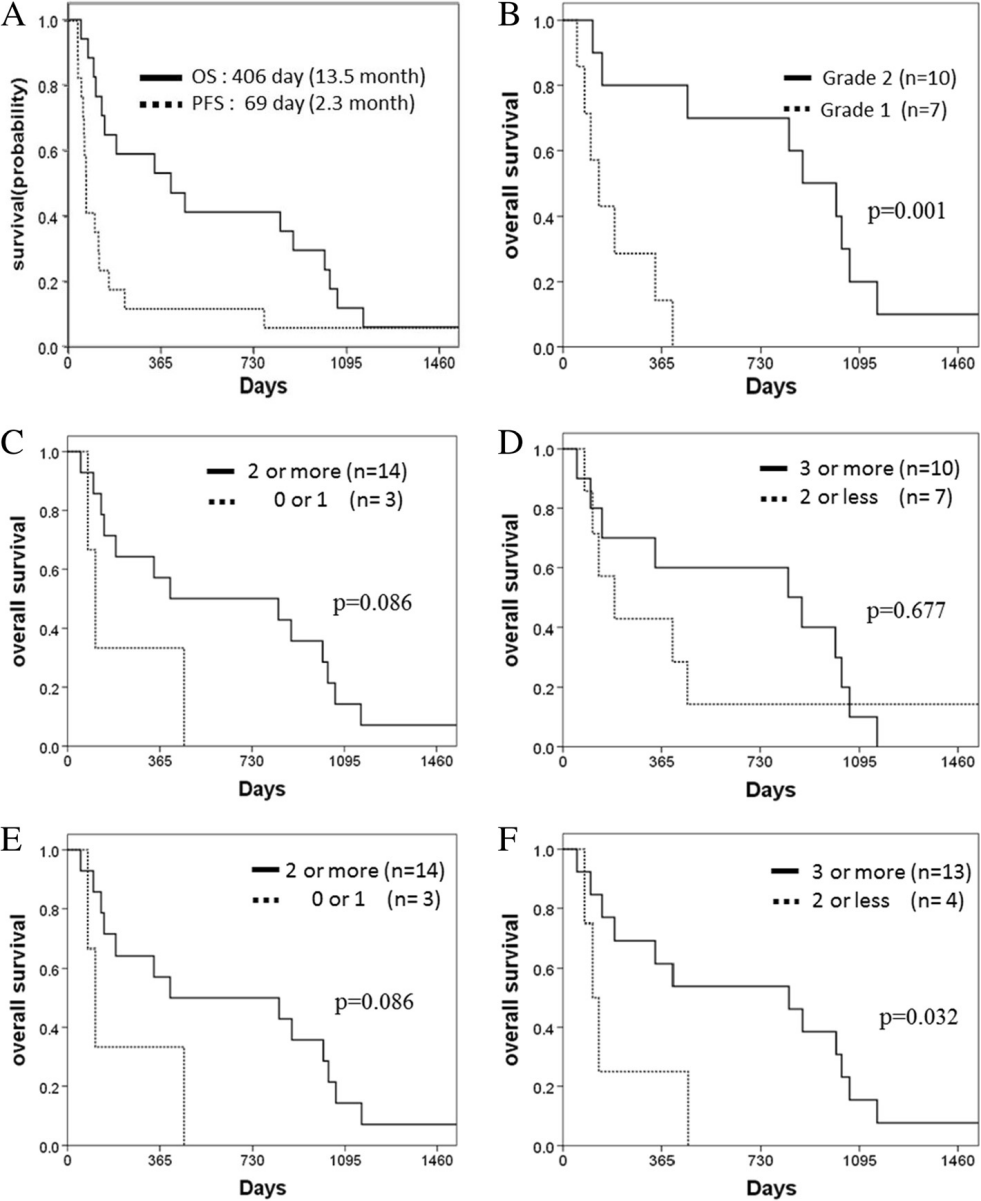
**Kaplan-Meier curves for survival of seventeen vaccinated patients. (A)** Overall survival (OS) and progression free survival (PFS) of seventeen vaccinated patients. **(B)** Overall survival according to the skin reactions. Local skin reactions at injected site of vaccine were assessed according to CTCAE grading. **(C, D)** Overall survival according to the number of peptide specific responses within 3 courses of the vaccination (C: 2 or more versus 1 or less, D: 3 or more versus 2 or less). **(E, F)** Overall survival according to the number of peptide specific responses within 6 courses of the vaccination (E: 2 or more versus 1 or less, F: 3 or more versus 2 or less).

Median PFS and OS periods of the 17 patients were 2.3 months (95% CI: 2.0-2.6) and 13.5 months (95% CI: 1.4-25.6), respectively (Figure 2A). The 2-year survival rate was 41.2% (95% CI: 17.9-64.5). Significant improvement of OS after 6 months from the first vaccination has implied the delayed response of the vaccination [24].

Patients with strong induration and redness, or those with ulceration (CTCAE grade2) survived significantly longer than patients without these reactions (Figure 2B). Patients with CTL responses to two or more peptides within 3 courses of the vaccination trended to have a longer survival (Figure 2C). Patients who revealed positive CTL responses against three or more peptides within 6 courses (24 weeks) of the vaccination showed a significantly longer survival (Figure 2F). In addition to the numbers of detected peptide specific T cell responses, strength of T cell responses such as +, ++, and +++ were evaluated in terms of efficacy of peptide vaccination and clinical outcome, for example, + versus ++ or more, or, ++ or less versus +++. There was no significant difference in the clinical outcome according to the strength of T cell responses (data not shown). Serum CEA level before treatment (more than 100 ng/ml or less) was also the predictive marker for the prognosis of these patients (p = 0.003, data not shown). Next we performed multivariate analysis of biomarkers for overall survival using Cox regression model (Table 4). For overall survival, multivariate analysis indicated that CTL responses to two or more peptides within 3 courses and strong skin reactions at injected site were significant predictors.

**Table 4:** Multivariate analysis of biomarkers for overall survival using Cox regression model

	**Variable**	**Hazard ratio**	**95% CI**	**P value**
Step 1	CEA: < 100 (ng/ml) versus ≥100	0.209	0.017 to 2.542	0.220
	Local skin reaction: grade2 versus grade1	0.171	0.013 to 2.190	0.175
	CTL response: 2 or more versus 1 or less (within 3 courses)	0.078	0.011 to 0.548	0.010
Step 2	Local skin reaction: garde2 versus garde1	0.048	0.007 to 0.326	0.002
	CTL response: 2 or more versus 1 or less (within 3 courses)	0.101	0.016 to 0624	0.014

CI, confidence interval.

## Discussion

We performed phase I study using five novel epitope peptides, including three peptides derived from three oncoantigens as well as two peptides targeting VEGFR1 and VEGFR2, for colorectal cancer. Vaccinations of five peptides in three different doses as well as cocktail treatment of five peptides in metastatic CRC patients were well tolerated without any severe systemic adverse events. Although two patients revealed ulceration at the skin injection site, they were able to continue the vaccine treatment. Hence, the safety of our study was confirmed as previous reports using the same class of peptides [21,22,25].

The average numbers of specific CTL induction against these five peptides after 12 vaccinations of the doses of 0.5 mg, 1.0 mg, and 3.0 mg were calculated to be 2.0, 2.3, and 3.2 peptides/patient, respectively. In this study, the maximum tolerated dose was not observed. Moreover, there was no sign of immunosuppression due to excessive administration at the doses of 3.0 mg. Although the number of the patients enrolled in this study was very limited, the higher dose of peptide seems to induce peptide-specific CTLs more effectively than the lower dose. Hence, we would like to the recommended dose of each peptide for further study to be 3.0 mg.

We also attempted vaccinations of the 5-peptide cocktail that was also well tolerated without any serious systemic toxicity. Although there were some concerns that the mixture of multiple peptides in a single injection might reduce the immune response due to the different affinity of each peptide to an HLA molecule, we observed that the average numbers of peptide-specific CTL inductions per patient was 3.5 within 3 courses of vaccinations. Our results demonstrated that the cocktail of multiple peptides is non-inferiority to the separate injection of each of multiple peptides. A previous report described that the frequency of CTL induction specific to vaccinated peptides in advanced CRC was approximately 33% [26]. However, we observed the peptide-specific CTL induction in more than 60% of the patients after the 12-week of vaccination at the dose of 3.0 mg/week, indicating that our peptide epitopes might have higher immunogenicity than peptides used previously.

Among the 18 patients we evaluated clinically, one patient showed the complete response and six patients kept stable conditions for 4–7 months. Hence, the response rate (RR) was calculated to be 5.6% and the disease control rate (DCR) to be 38.9%. One review article reported that the RR of active cancer vaccines for CRC was 0.9% and the DCR was 11.1% [26]. Hence, our clinical data has also implied the possible higher immunogenicity of our vaccines.

Although our peptide vaccination did not show obvious superiority in the aspect of RR or DCR, compared to the worldwide standard therapies for CRC [27,28], the median overall survival time (MST) of our patients was 13.5 months which seemed longer than other presently-available standard therapies; for example, the MST was 6.1 months and 6.2 months in a phase III trial of Cetuximab [27] or Panitumumab [28] in patients with chemotherapy-refractory metastatic CRC, respectively. As US Food and Drug Administration described in “The guidance for therapeutic cancer vaccine”, this kind of vaccine treatment usually takes a few to several months to show clinical benefit due to the time lag to induce the sufficient number of effecter cells and is expected to demonstrate delayed effects [24]. The relation between peptide specific responses and OS was shown in Figure 2. Patients who revealed the CTL induction to two or more peptides within 3 courses (MST: 13.5 months) trended to survive longer (P = 0.086) than patients with CTL induction to one or no peptide (MST: 3.7 months). Moreover, patients with CTL induction against three or more peptides within 6 courses of the vaccination (MST: 27.8 months) had significantly longer survival (P = 0.032) than the remaining patients (MST: 3.7 months), suggesting the more CTLs were induced, the better prognosis was expected and supporting the use of multiple peptides for advanced cancer patients. Similarly, local reactions at peptides-injected sites could be possibly a good predictive biomarker(s) for longer survival; Patients with grade 2 local skin reactions at injected site of vaccine survived significantly (P = 0.001) longer (MST: 29.5 months) than patients with grade 1 reactions (MST: 4.4 month). These data suggested that the monitoring of the CTL responses and the skin reactions might become good predictive markers during the treatment for the efficacy of vaccination. Although it is very difficult at present, the prior selection of patients who are likely to respond well and induce CTLs effectively to vaccination is also very important. The presence of a higher number of infiltrated T cells in tumor microenvironment was suggested as a predictive biomarker for the response to immunotherapies and the selection of patients with the better treatment outcome of vaccinations [29].

In conclusion, although the number of patients in this early-phase trial is very limited, our peptides vaccine therapy was demonstrated to be safe, effectively induce peptide-specific immune responses and possibly improve the prognosis of advanced colorectal cancer. It is certain that although we need to verify this preliminary result by a much larger double-blind study, we believe that further stages of clinical trials should be worth doing.

## Conclusions

This study indicated that the combination of five novel peptides can induce strong peptide specific immune responses in the group who received the dosage of 3.0 mg, and that the single injection of “the peptide cocktail” is likely to induce the similar immune responses to the multiple injections of “each peptide”, and that the overall survival of patients treated with our peptides was prolonged obviously after 6 months from the first vaccination, which has implied the delayed response of the vaccination. Moreover, the induction of peptide specific immune responses had significant relevance to longer survival. Although we need to verify this preliminary result by a much larger double-blind study, we believe that these findings surely lead to the novel therapeutic strategy for advanced colorectal cancer.

## Abbreviations

RNF43: Ring finger protein 43; TOMM34: 34 kDa-translocase of the outer mitochondrial membrane; KOC1: Insulin-like growth factor–II mRNA binding protein 3; VEGFR: Vascular endothelial growth factor receptor; CRC: Colorectal cancer; ELISPOT: Enzyme-linked immunospot; PBMC: Peripheral blood mononuclear cells; CTL: Cytotoxic T lymphocytes; CR: Complete clinical response; SD: Stable disease; PD: Progressive disease; PFS: Progression free survival; OS: Overall survival; HLA: Human leukocyte antigen; MST: Median overall survival time; ECOG: Eastern cooperative oncology group; RECIST: Response evaluation criteria in solid tumors; TIL: Tumor infiltrating cells; CTCAE: Common Terminology Criteria for Adverse Events version3.0; PS: Performance status; HPLC: high performance liquid chromatography; HIV-Env: The human immunodeficiency virus-envelope; IFA: Incomplete Freund's adjuvant; CT: Computed tomography; MRI: Magnetic resonance imaging; IFN: Interferon.

## Competing interests

Yusuke Nakamura is a stock holder and a scientific advisor of OncoTherapy Science, Inc. The other authors have no potential conflicts of interest to disclose.

## Authors’ contributions

SH designed, performed and evaluated clinical study, and wrote the manuscript. YN and MO participated in the design, review and revision of the manuscript. KT participated as the main coordinator and investigator regarding the immunological data analysis and evaluation. HT, NS, RT, YI YT, NI, SY, HS, AK, and HF assisted to perform clinical study and data analysis. All authors participated in the data acquisition and discussion of the manuscript and approved the final manuscript.

## Supplementary Material

Additional file 1: Figure S1 Positivity of antigen-specific T cell response was quantitatively defined according to the evaluation tree algorithm. In brief, the peptide-specific spots (SS) were the average of triplicates by subtracting the HIV peptide-pulsed stimulator well from the immunized peptide-pulsed stimulator well. The %SS means the percentage of SS among the average spots of the immunized peptide pulsed stimulator well. The positivity of antigen-specific T cell response were classified into four grades (−, +, ++, and +++) depending on the amounts of peptide-specific spots and invariability of peptide-specific spots at different responder/stimulator ratios. SS, peptide-specific spots; R1, responder/stimulator ratio = 1; R2, responder/stimulator ratio = 0.5; R3, responder/stimulator ratio = 0.25; R4, responder/stimulator ratio = 0.125. Click here for file

Additional file 2: Figure S2 Representative immunologic monitoring assays detecting antigen-specific T-cell responses in patient 10 **(A)** and 16 **(B, C)**, which were induced interferon-g (IFN-g)-producing cells. Positivity of antigen-specific T-cell response was quantitatively defined according to the evaluation tree algorithm (Additional file 1: Figure S1). Click here for file
