# Supplementary material for: A phase I study of combination vaccine treatment of five therapeutic epitope-peptides for metastatic colorectal cancer; safety, immunological response, and clinical outcome
Source: J Transl Med. 2014 Mar 10;12:63. doi: 10.1186/1479-5876-12-63 (PMC4007571; doi:10.1186/1479-5876-12-63)
Supplement: Additional file 1: Figure S1 — Positivity of antigen-specific T cell response was quantitatively defined according to the evaluation tree algorithm. In brief, the peptide-specific spots (SS) were the average of triplicates by subtracting the HIV peptide-pulsed stimulator well from the immunized peptide-pulsed stimulator well. The %SS means the percentage of SS among the average spots of the immunized peptide pulsed stimulator well. The positivity of antigen-specific T cell response were classified into four grades (−, +, ++, and +++) depending on the amounts of peptide-specific spots and invariability of peptide-specific spots at different responder/stimulator ratios. SS, peptide-specific spots; R1, responder/stimulator ratio = 1; R2, responder/stimulator ratio = 0.5; R3, responder/stimulator ratio = 0.25; R4, responder/stimulator ratio = 0.125. [file 1479-5876-12-63-S1.pdf]

Supplemental Figure 1

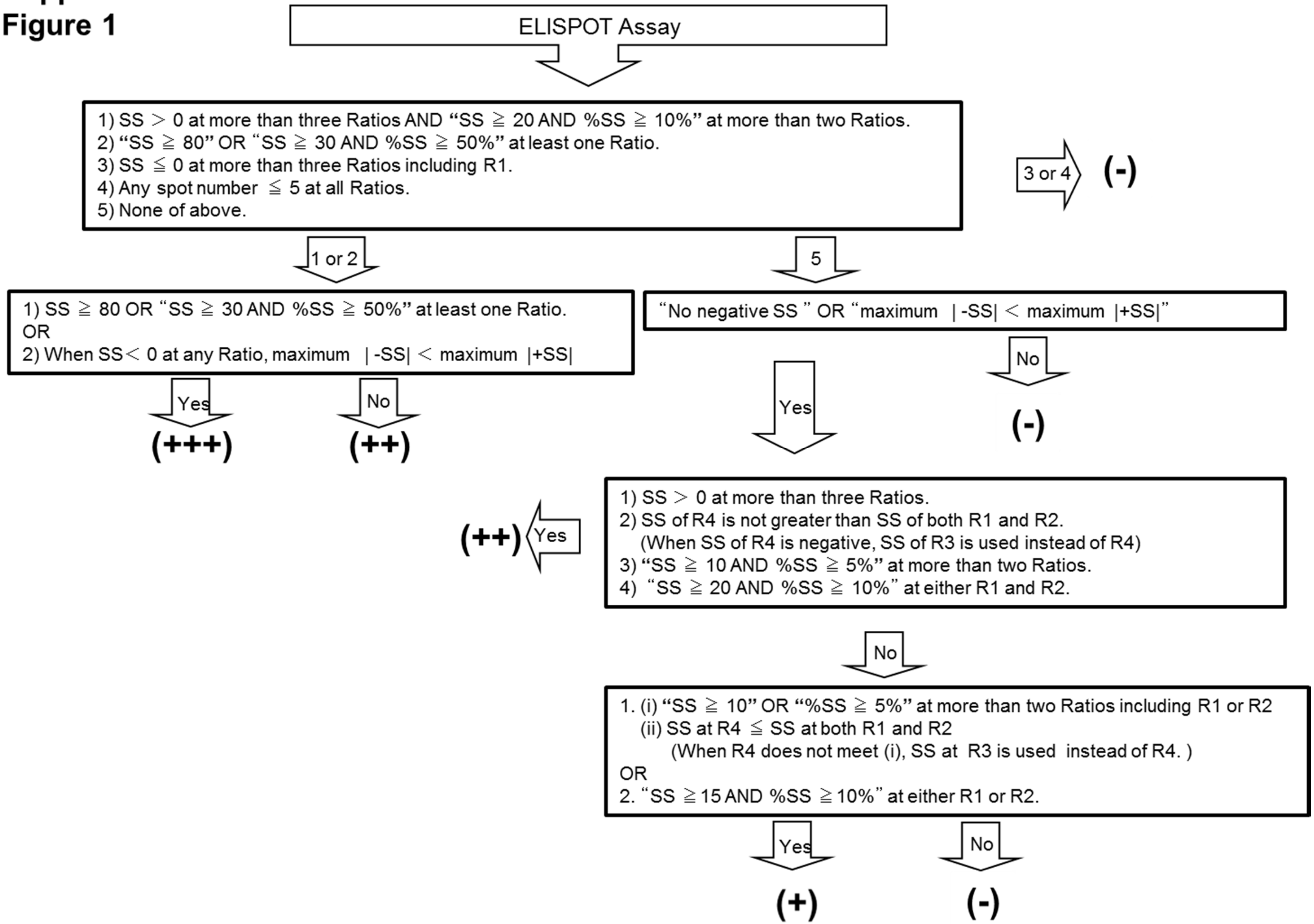

Figure S1. Positivity of antigen-specific T cell response was quantitatively defined according to the evaluation tree algorithm.
In brief, the peptide-specific spots (SS) were the average of triplicates by subtracting the HIV peptide-pulsed stimulator well from the immunized peptide-pulsed stimulator well. The %SS means the percentage of SS among the average spots of the immunized peptide pulsed stimulator well. The positivity of antigen-specific T cell response were classified into four grades (ī , +, ++, and +++) depending on the amounts of peptide-specific spots and invariability of peptide-specific spots at different responder/stimulator ratios.
SS, peptide-specific spots; R1, responder/stimulator ratio = 1; R2, responder/stimulator ratio = 0.5; R3, responder/stimulator ratio = 0.25; R4, responder/stimulator ratio = 0.125.
