# Supplementary material for: A phase I study of combination vaccine treatment of five therapeutic epitope-peptides for metastatic colorectal cancer; safety, immunological response, and clinical outcome
Source: J Transl Med. 2014 Mar 10;12:63. doi: 10.1186/1479-5876-12-63 (PMC4007571; doi:10.1186/1479-5876-12-63)
Supplement: Additional file 2: Figure S2 — Representative immunologic monitoring assays detecting antigen-specific T-cell responses in patient 10 (A) and 16 (B, C), which were induced interferon-g (IFN-g)-producing cells. Positivity of antigen-specific T-cell response was quantitatively defined according to the evaluation tree algorithm (Additional file 1: Figure S1). [file 1479-5876-12-63-S2.pdf]

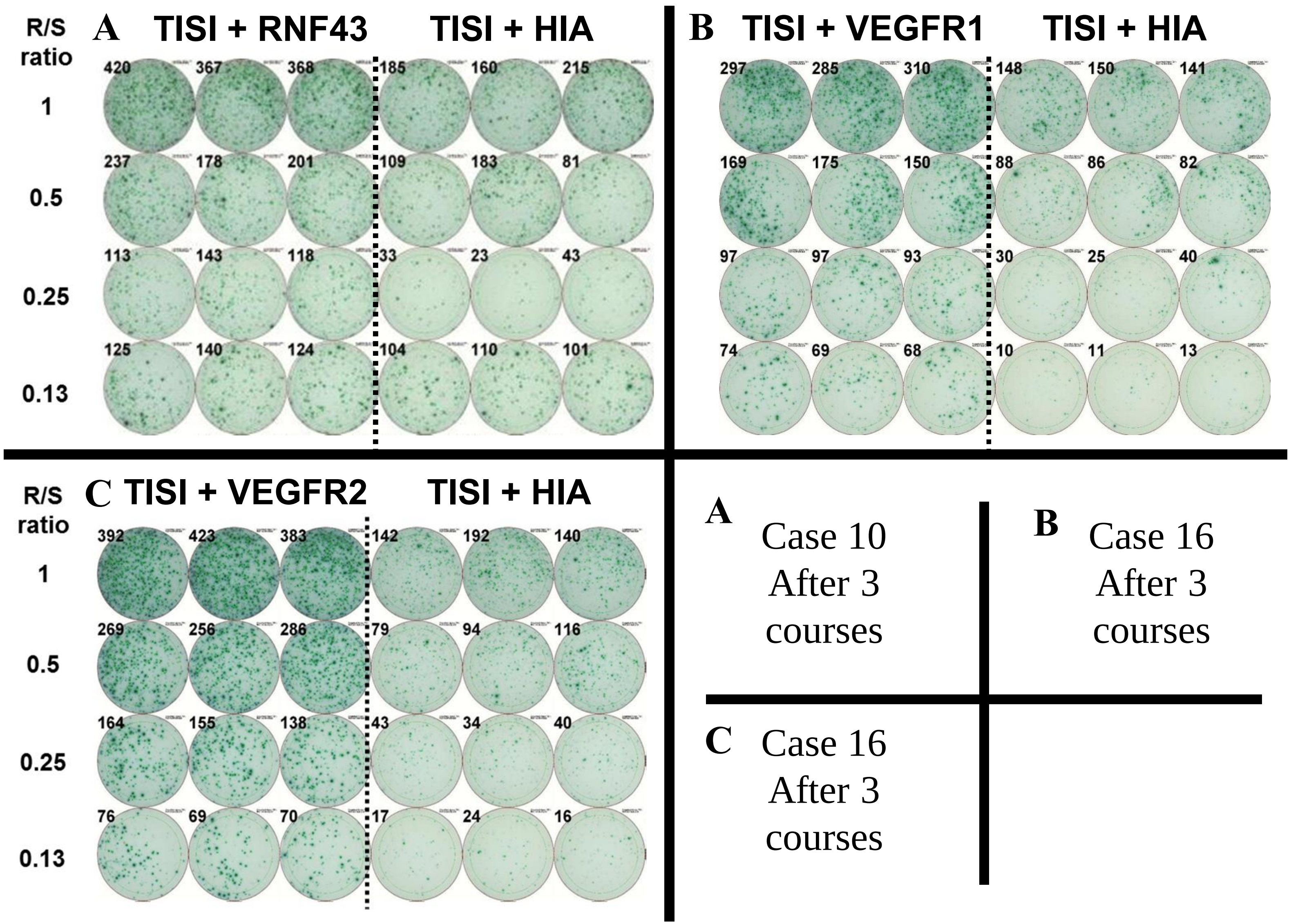

Supplemental Figure 2.
Representative immunologic monitoring assays detecting antigen-specific T-cell responses in patient 10 (A) and 16 (B, C), which were induced interferon-γ (IFN-γ)-producing cells. Positivity of antigen-specific T-cell response was quantitatively defined according to the evaluation tree algorithm (Supplemental Figure 1).
